# Comparative Proteomic Analysis of Wild-Type *Physcomitrella Patens* and an OPDA-Deficient *Physcomitrella Patens* Mutant with Disrupted *PpAOS1* and *PpAOS2* Genes after Wounding

**DOI:** 10.3390/ijms21041417

**Published:** 2020-02-19

**Authors:** Weifeng Luo, Setsuko Komatsu, Tatsuya Abe, Hideyuki Matsuura, Kosaku Takahashi

**Affiliations:** 1 Division of Fundamental Agroscience Research, Research Faculty of Agriculture, Hokkaido University, Kita 9, Nishi 9, Kita-ku, Sapporo 060-8589, Japan; luoweifeng1989@outlook.com (W.L.); abet@nitten.co.jp (T.A.); matsuura@hokudai.ac.jp (H.M.); 2 Department of Environmental and Food Sciences, Faculty of Environmental and Information Sciences, Fukui University of Technology, 3-6-1 Gakuen, Fukui 910-8505, Japan; skomatsu@fukui-ut.ac.jp; 3 Department of Nutritional Science, Faculty of Applied Bioscience, Tokyo University of Agriculture, 1-1-1 Sakuragaoka, Setagaya-ku, Tokyo 165-8502, Japan

**Keywords:** Allene oxide synthase, 12-oxo-phytodienoic acid, *Physcomitrella patens*, proteomic analysis, wounding

## Abstract

Wounding is a serious environmental stress in plants. Oxylipins such as jasmonic acid play an important role in defense against wounding. Mechanisms to adapt to wounding have been investigated in vascular plants; however, those mechanisms in nonvascular plants remain elusive. To examine the response to wounding in *Physcomitrella patens*, a model moss, a proteomic analysis of wounded *P. patens* was conducted. Proteomic analysis showed that wounding increased the abundance of proteins related to protein synthesis, amino acid metabolism, protein folding, photosystem, glycolysis, and energy synthesis. 12-Oxo-phytodienoic acid (OPDA) was induced by wounding and inhibited growth. Therefore, OPDA is considered a signaling molecule in this plant. Proteomic analysis of a *P. patens* mutant in which the *PpAOS1* and *PpAOS2* genes, which are involved in OPDA biosynthesis, are disrupted showed accumulation of proteins involved in protein synthesis in response to wounding in a similar way to the wild-type plant. In contrast, the fold-changes of the proteins in the wild-type plant were significantly different from those in the *aos* mutant. This study suggests that *PpAOS* gene expression enhances photosynthesis and effective energy utilization in response to wounding in *P. patens.*

## 1. Introduction

The influence of abiotic and biotic stresses on plants has been particularly well studied [1]. Wounding is an abiotic stress that can cause severe damage to plants. Wounded tissues are more likely to be infected by pathogenic microorganisms, which cause serious damage to plants. However, to protect cells from irreversible damage caused by wounding, plants induce changes in the abundance of many proteins, indicating that plants have developed resistance mechanisms against stresses, including wounding.

Plant adaptations against wounding have been extensively investigated. Various responses to wounding have been elucidated, including activation of metabolic pathways, cell-wall modifications, and the production of pathogenesis-related proteins and proteinase inhibitors [2,3]. Moreover, plant stress hormones play a critical role in the defense against wounding. Jasmonic acid (JA) is among the most important signaling hormones in response to wounding in vascular plants [4]. A recent study indicated that JA also functions as a signaling compound in the model lycophyte *Selaginella moellendorffii* [5].

JA, which is synthesized from α-linolenic acid through the octadecanoid pathway, modulates the expression of various genes in response to abiotic and biotic stresses [4,6,7]. The JA signaling pathway is known in detail. The isoleucine conjugate of JA (JA-Ile) is a versatile compound in the JA signaling pathway [8,9,10,11]. The binding of JA-Ile with its receptor coronatine insensitive 1 (COI1) triggers various physiological responses in plants. 12-Oxo-phytodienoic acid (OPDA), an intermediate of JA biosynthesis, shows JA-dependent and JA-independent biological activities in plants [12,13,14,15,16]. OPDA induces expression of a set of genes in response to wounding in *Arabidopsis thaliana* and plays important roles in embryo development and seed germination in tomato [17,18,19,20]. However, a detailed OPDA signaling mechanism remains elusive in plants.

Bryophytes, including *Marchantiophyta* (liverworts), *Bryophyta* (mosses), and *Anthocerotophyta* (hornworts), are taxonomically positioned between algae and vascular plants and comprise an early-diverging lineage of land plants [21,22]. Therefore, bryophytes occupy a key evolutionary position and aid in our understanding of the molecular basis of the key innovations that allowed green plants to evolve from aquatic ancestors and adapt to the terrestrial environment [22]. *Physcomitrella patens*, a model moss, has been utilized to study basic plant physiology and development, as well as the molecular mechanisms of plant evolution from the hydrosphere to land [23]. The life cycle of *P. patens* is characterized by two generations: a haploid gametophyte and a diploid sporophyte. A spore develops into a filamentous structure called the protonema, which can differentiate into structurally complex gametophores with leaf-like structures, rhizoids, and the sexual organ [24]. As the protonema is distinctly different from the gametophore, the expression profiles of the genes and proteins in the protonema are different from those of the gametophore. 

Transcriptomic and proteomic analyses have been conducted to understand the molecular basis of environmental stress tolerance in *P. patens* [25,26,27,28]. Specifically, drought tolerance studies in *P. patens* could help to elucidate how plants acquired drought resistance, thereby permitting their invasion of the terrestrial environment. However, the influence of wounding, a severe environmental stress in plants, has been overlooked in *P. patens* until recently. Previous research found that OPDA and methyl jasmonate (MeJA), but not JA, retards the growth of *P. patens* [29]. Unlike vascular plants, *P. patens* produces OPDA, but not JA, in response to wounding and pathogenic infection [29,30]. These findings strongly suggest that the first half of the octadecanoid pathway in chloroplasts is conserved in *P. patens* and that OPDA, not JA, functions as a signaling molecule in *P. patens*. The differences in the responses to JA and OPDA between vascular plants and bryophytes are a significant observation in the study of plant evolution. Moreover, understanding the functions of OPDA in *P. patens* would help to elucidate the OPDA signal transduction pathway in plants. Allene oxide synthase (AOS) is an OPDA biosynthetic enzyme. AOS converts 13(*S*)-hydroperoxyoctadecatrienoic acid (13-HPOT) to 12,13(*S*)-epoxyoctadecatrienoic acid (12,13-EOT), and this product is then cyclized by allene oxide cyclase (AOC) into OPDA. Two AOS genes, *PpAOS1* and *PpAOS2*, are present in the genome of *P. patens* [31]. To investigate the mechanism underlying the adaptation to wounding and the role of AOS gene expression leading to OPDA synthesis in the response to wounding, we used wild-type *P. patens* and an OPDA-deficient mutant with disrupted *PpAOS1* and *PpAOS2* in this research. For analysis of the mechanism underlying the adaptation to wounding of *P patens*, a gel-free/label-free proteomic technique was used. Furthermore, gene expression analysis was performed to confirm the proteins identified by proteomic analysis.

## 2. Results and Discussion

### 2.1. Generation of 12-Oxo-phytodienoic acid (OPDA)-deficient P. Patens Mutants with Disrupted PpAOS1 and PpAOS2 Genes

**Figure 1:**
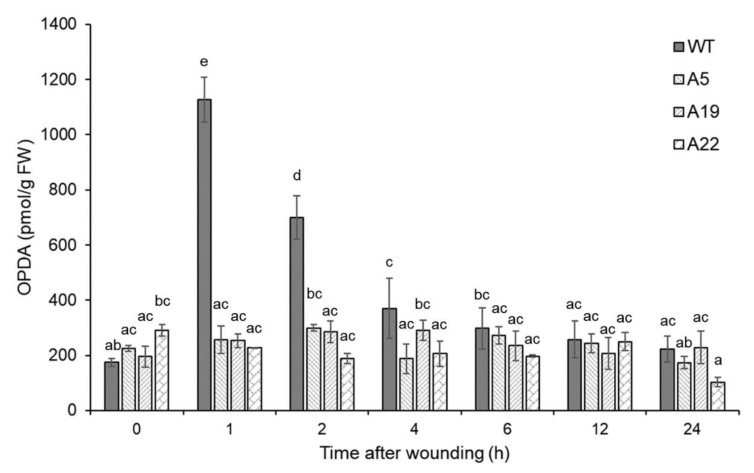
Accumulation of 12-oxo-phytodienoic acid (OPDA) in *P. patens* after wounding. The wild-type and *aos* mutants of *P. patens* were grown on BCDAT agar for 3 weeks and then treated with wounding. The samples were prepared at 1, 2, 4, 6, 12, and 24 h after wounding. Three independent experiments were conducted as biological replicates. The concentrations of OPDA in the wild-type and *aos* mutants (A5, A19, and A22) after wounding were analyzed using ultra-performance liquid chromatography-tandem mass spectrometry (UPLC-MS/MS). The values are the mean ± SD (*n* = 3). Different letters indicate that the change is significant as determined by one-way ANOVA according to Tukey’s multiple comparison test (*p* < 0.05).

**Figure 2:**
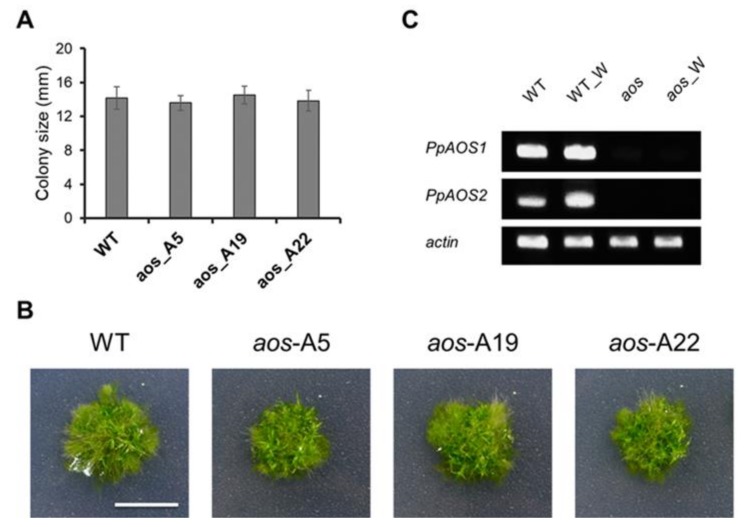
Phenotypic analysis of the wild-type and *aos* mutant of *P. patens*. The wild-type and the *aos* mutants of *P. patens* were grown on BCDAT agar for 3 weeks, and their phenotypes were compared. (**A**) Colony size of the wild-type and *aos* mutants of *P. patens* (the values are the mean ± SD, *n* = 12). A significant difference was not found between wild-type and mutants (Student’s t-test). (**B**) Images of the wild-type and *aos* mutants of *P. patens*. White size bar represents 1 cm length. (**C**) Semi-quantitative RT-PCR of *PpAOS* gene expression after wounding in the wild-type and *aos* mutant (A5 strain). WT and aos as control, without wounding treatment; WT_W and aos_W as wounded group: gametophores of *P. patens* (WT and *aos* mutant) were wounded using tweezers and harvested 1 h later for RNA isolation. The gels were stained using ethidium bromide.

To study the influence of *AOS* gene expression in physiology of *P. patens*, we generated three *P. patens* OPDA-deficient mutants in which both *PpAOS1* and *PpAOS2* were disrupted (A5, A19, and A22). An ultra-performance liquid chromatography-tandem mass spectrometry (UPLC-MS/MS) analysis of OPDA in the three mutants revealed that wounding did not induce OPDA accumulation (Figure 1). Gametophores of these three double-knockout mutants were grown for three weeks under standard conditions, which led to the formation of colonies (Figure 2, Figure S1). Compared to wild-type, we did not observe any differences in growth in the double-knockout mutants, which was similar to a previous report of a single knockout mutant [31].

The OPDA concentration in these mutants was lower than that in the wild-type in the first 2 h after wounding; however, the disruption of these two *PpAOS* genes did not completely prevent OPDA synthesis (Figure 1). Because hydroperoxide lyases (PpHPLs) show weak AOS activities in *P. patens* [31], it is possible that the OPDA detected in the mutants was produced by PpHPLs. In a previous study, the disruption of *PpAOC1* and *PpAOC2* genes caused reduced fertility, aberrant sporophyte morphology and interrupted sporogenesis in *P. patens* [13]. However, the phenotype of the *PpAOS1* and *PpAOS2* mutants was not significantly different from that of wild-type under conventional growth conditions (Figure 2), indicating that the trace amount of OPDA detected in the mutants was apparently sufficient for the growth of *P. patens*.

As the phenotypes of the *P. patens* mutants in which *PpAOS1* and *PpAOS2* were disrupted (A5, A19, and A22) were almost the same, the A5 strain (referred to as the *aos* mutant hereafter) was utilized to investigate the influence of *PpAOS* gene disruption at the protein level in response to wounding.

### 2.2. Identification of Proteins that are Differentially Accumulated in Response to Wounding

To identify *P. patens* proteins that are altered by wounding, gel-free/label-free proteomic analysis was performed. The outline of the procedure used for proteomic analysis in this study is illustrated in Figure S2. In reports of investigation of plant stress adaptation, samples were collected at each time point after stress (30 min to several days) [32]. A certain period is required for signal transduction and protein synthesis after stress. Twenty-four hours is one of the typical time points to analyze stress responses for proteome analyses. Thus, proteins were extracted from 3-week-old wild-type *P. patens* and the *aos* mutant at 24 h after wounding. The extracted proteins were digested, and the resulting peptides were analyzed using nano LC-MS/MS [33]. Three biological replicates were utilized in this study. The levels of 136 and 88 proteins with more than two matched peptides were significantly altered by more than 1.5-fold in response to wounding in the wild-type and the *aos* mutant, respectively (*p* < 0.05) (Tables S1 and S2). Wounding increased the abundance of 114 proteins in the wild-type and 88 proteins in the *aos* mutant compared with untreated plants, while only 22 proteins in the wild-type decreased due to wounding. Remarkably, none of the proteins in the *aos* mutant showed a significant decrease in abundance in response to wounding. It appears that *PpAOS* gene expression is related to the decrease in protein abundance observed in response to wounding.

### 2.3. Functional Categories of Identified Wounding-Responsive Proteins

**Figure 3:**
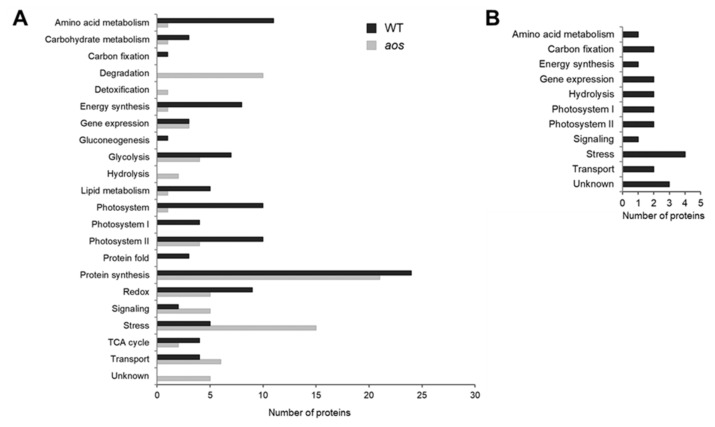
Functional categorization of proteins identified in response to wounding stress in *P. patens* wild-type and the *aos* mutant. (**A**) Functional categorization of proteins that accumulated in response to wounding in *P. patens* wild-type and the *aos* mutant. (**B**) Functional categorization of proteins that decreased in response to wounding in the wild-type *P. patens*. Three-week-old *P. patens* tissues were wounded using tweezers. After 24 h, proteins were extracted from the tissue and analyzed using a gel-free/label-free proteomic technique, and significantly changed proteins (*p* < 0.05, matched peptides >2) were identified using a student’s *t*-test. The identified proteins were annotated using Phytozome ver. 11.0.9 (https://phytozome.jgi.doe.gov/pz/portal.html) and functionally categorized.

To determine the functions of the identified proteins, the proteins were annotated using Phytozome (https://phytozome.jgi.doe.gov/pz/portal.html) and functionally categorized (Figure 3). To investigate the functional relationship of the proteins identified in response to wounding in *P. patens* (wild-type and *aos* mutant), significantly enriched Gene Ontology (GO) terms and Kyoto Encyclopedia of Genes and Genomes (KEGG) pathways were identified according to the *P* value and enrichment factor [34]. Proteins were significantly altered by wounding in wild-type and/or the *aos* mutant (Figure 3). In the wild-type plant, wounding predominantly increased the abundance of proteins related to photosystems, protein synthesis, amino acid metabolism, redox, and energy synthesis. The *aos* mutant largely accumulated proteins related to protein synthesis, stress, and protein degradation in response to wounding.

**Figure 4:**
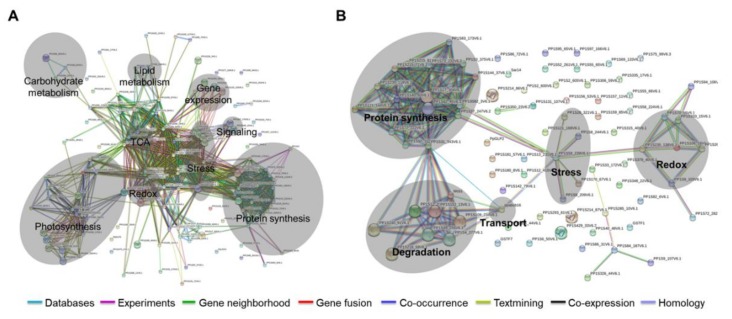
The Search Tool or the Retrieval of Interacting Genes (STRING) bioinformatic analysis of significantly accumulated proteins in *P. patens* after wounding. (**A**) Visualization of the protein interaction network of significantly differentially accumulated proteins in wild-type *P. patens* after wounding. The shaded analysis networks are for photosynthesis, carbohydrate metabolism, lipid metabolism, TCA, redox, stress, gene expression, signaling, and protein synthesis. Databases and text-mining were chosen as the active prediction methods. (**B**) Visualization of the protein interaction network of significantly differentially accumulated proteins in the *aos* mutant after wounding. The shaded analysis networks are for protein synthesis, degradation, transport, stress, and redox. Databases and text-mining were chosen as the active prediction methods.

To further analyze the correlations of the differentially expressed proteins, protein–protein interactions were determined, and pathway prediction was performed. The Search Tool or the Retrieval of Interacting Genes (STRING) database was used to calculate all direct interactions between the 136 and 88 proteins identified in wild-type and the *aos* mutant. As shown in Figure 4, a complicated network of protein–protein interactions were found in the wild-type. The proteins categorized in each group were closely related each other. In contrast to the wild-type plant, the interactions between each protein group, which were clustered based on functions, were remotely related in the *aos* mutant. Comparison of *aos* mutant and WT suggested that fewer proteins interacted in the *aos* mutant due to wounding.

### 2.4. Subcellular Localization of the Identified Proteins in Response to Wounding

**Figure 5:**
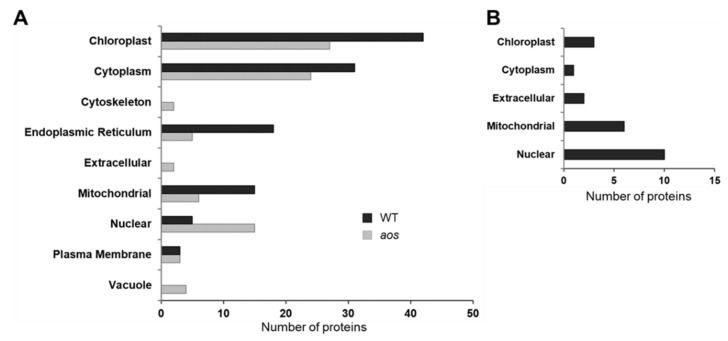
Subcellular localization of proteins that changed in abundance in response to wounding in *P. patens* wild-type and the *aos* mutant. (**A**) Increased proteins. (**B**) Decreased proteins. The subcellular localization of the identified proteins was predicted using TargetP, Bacello, and WoLF PSORT.

To predict the subcellular localization of the proteins whose abundance was altered in the wild-type and the *aos* mutant under wounding stress, we conducted bioinformatic analysis [34]. Subcellular localization data revealed that more than 30% of the proteins with changes in abundance in response to wounding, including 52 proteins in the wild-type and 27 proteins in the *aos* mutant, were predicted to be localized to the chloroplast (Figure 5). Moreover, 15 proteins with increased abundance were predicted to be localized to the nucleus in the *aos* mutant, and 8 proteins were inferred to be localized to the nucleus in the wild-type. The bioinformatic findings suggested that proteins in chloroplasts play an important role in the physiological response to wounding in *P. patens.*

### 2.5. Protein Catalogs of the Identified Proteins

#### 2.5.1. Proteins Involved in Protein Synthesis

A considerable number of proteins involved in protein synthesis increased in response to wounding in the wild-type plant (Figure 3, Table S1). Ribosomal proteins were mainly accumulated by wounding. The number of ribosomal proteins that accumulated in the *aos* mutant was comparable to that in the wild-type. These data indicated that *AOS* gene disruption was not directly related to the accumulation of proteins involved in protein synthesis.

The representative physiological reactions to wounding in plants include increases in cell-wall integrity, secondary metabolite synthesis, and stress-related plant hormone synthesis [35]. Furthermore, many metabolic pathways are activated in response to wounding. Before the induction of adaptive responses to wounding stress, the de novo production of proteins associated with those physiological events is required [35]. Therefore, increasing the abundance of proteins involved in protein synthesis is a significant physiological response in the adaptation to wounding in *P. patens*.

#### 2.5.2. Proteins Involved in Protein Degradation

Interestingly, the levels of 10 identified proteins related to protein degradation (26S proteasome-related proteins) were increased in the *aos* mutant (Figure 3, Table S2). In contrast, increased abundance of proteins involved in protein degradation was not observed in the wild-type plant. Proteasomes are protein complexes that are related to protein degradation, which plays a major role in regulating cell physiology [36,37]. Because the increase in the 26S proteasome-related proteins was observed only in the *aos* mutant, *PpAOS* gene expression is suggested to suppress the accumulation of 26S proteasome-related proteins in the wild-type plant.

#### 2.5.3. Proteins Involved in Amino Acid Metabolism

The abundance of more than 10 proteins related to amino acid metabolism was increased after wounding in the wild-type plant, including ketol-acid reductoisomerase, acetohydroxy acid isomeroreductase, dihydropyrimidine dehydrogenase, pyridoxal phosphate-dependent enzyme synthase, vitamin-B12 independent methionine 5-methyltetrahydropteroyltriglutamate-homocysteine, glutamate dehydrogenase, and amino acid binding protein (Figure 3, Table S1). In contrast, only one protein, asparagine synthetase, was accumulated in the *aos* mutant in response to wounding (Figure 3, Table S2). The number of proteins involved in amino acid synthesis whose abundance was increased was substantially greater in the wild-type than in the *aos* mutant. *PpAOS* gene expression is suggested to enhance the synthesis of amino acids in response to wounding. Amino acids are the basic units of proteins and are thus required for protein synthesis. It is considered that simultaneous increases in the abundance of proteins related to both amino acid production and protein synthesis is advantageous in efficient and adequate protein synthesis for stress adaptation.

#### 2.5.4. Proteins Involved in Protein Folding

Proteins involved in the correct folding of other proteins, such as heat shock proteins (HSPs), which are classified as stress proteins in this report, accumulated in both the wild-type and the *aos* mutant of *P. patens* after wounding (Figure 3, Tables S1 and S2). These proteins are involved in spatial structural changes in a protein, which can trigger signaling that activates and/or deactivates a function [38]. Chaperonins, which are important for correct protein folding, were shown to be the highest-abundance proteins in only wild-type and were the most abundant among the identified wound-induced proteins (Table S1) [39]. Wound-induced accumulation of chaperonins in *P. patens* is inferred to depend on *PpAOS* expression. In contrast to the chaperonins detected in the wild-type plant, late embryogenesis abundant (LEA) proteins accumulated in the *aos* mutant (Table S2). LEA proteins were reported to suppress protein aggregation, which is promoted by abiotic stresses [40]. LEA proteins may be involved in protein folding in the *aos* mutant subjected to wounding.

As protein synthesis is activated in response to wounding stress, correct protein folding in wounded plants is more important than in untreated plants. This study revealed wound-stimulated increases in proteins involved in protein folding, accompanied by enhanced protein synthesis. Increased protein synthesis could require more proteins related to protein folding in wounded *P. patens.*

#### 2.5.5. Proteins Involved in Photosystems

**Figure 6:**
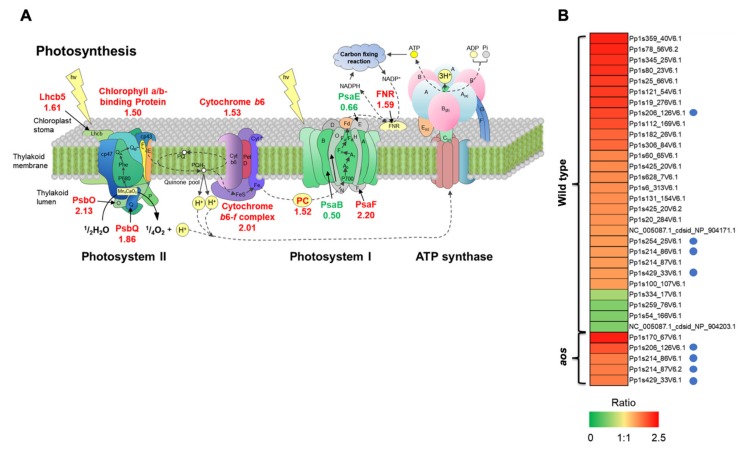
Differentially accumulated proteins involved in photosynthesis. (**A**) Proteins involved in photosynthesis were differentially accumulated in response to wounding stress in wild-type *P. patens*. Red and green numbers represent the relative abundance of proteins related to photosynthesis in response to wounding in the wild-type. Lhcb5 (Pp1s628_7V6.1, Pp1s6_313V6.1), light-harvesting complex II protein Lhcb5; chlorophyll a/b-binding protein (Pp1s214_86V6.1, Pp1s214_87V6.1, Pp1s429_33V6.1), type III chlorophyll a/b-binding protein; cytochrome b6 (NC_005087.1_cdsid_NP_904171.1), cytochrome b6; PsbO (Pp1s25_66V6.1, Pp1s306_84V6.1, Pp1s60_65V6.1), photosystem II manganese-stabilizing protein PsbO; PsbQ (Pp1s182_26V6.1), photosystem II oxygen-evolving complex protein PsbQ; cytochrome b6f complex (Pp1s112_169V6.1), cytochrome b6-f complex iron-sulfur subunit; PC (Pp1s254_25V6.1), plastocyanin; PsaB (NC_005087.1_cdsid_NP_904203.1), photosystem I P700 chlorophyll a apoprotein A2 (PsaB); PsaF (Pp1s345_25V6.1, Pp1s80_23V6.1, Pp1s121_54V6.1, Pp1s19_276V6.1), photosystem I reaction center protein PsaF; PsaE (Pp1s334_17V6.1), photosystem I reaction center subunit IV (PsaE); FNR (Pp1s131_154V6.1), ferredoxin-NADP+ reductase. (**B**) Heat map represents the profile of differentially accumulated photosystem-related proteins induced by wounding in *P. patens*. Red indicates high accumulation, whereas green indicates low accumulation. Blue circles indicate proteins that accumulated in both wild-type and the *aos* mutant.

Proteins involved in photosystems were a major protein group induced by wounding in the wild-type plant (Figure 3 and Figure 6, Table S1), including the photosystem I reaction center protein PsaF subunit III, the photosystem II manganese-stabilizing protein PsbO, oxygen-evolving enhancer protein I, light-harvesting complex II protein lhcb5 (chlorophyll a/b-binding protein), type III chlorophyll a/b-binding protein, and the small subunit of Rubisco.

Photosynthesis, which is among the most important plant physiological processes, converts light energy to chemical energy and produces carbohydrates, such as sucrose and starch, from water and carbon dioxide using light energy. The activation of the defense system, including protein synthesis, requires additional energy in plants [35]. Wounding is suggested to result in the accumulation of proteins involved in photosystems to provide energy for various stress responses. This study indicated that wounding induced the accumulation of fewer photosystem proteins in the *aos* mutant than in the wild-type. These results are accordance with the reported accumulation of proteins related to photosystems in OPDA-treated *P. patens* [30,41]. The genes encoding the photosystem proteins showing the greatest increases in response to wounding are present in the chloroplast genome. As OPDA is biosynthesized through AOS reaction in chloroplasts [4], OPDA may be important to regulate gene expression in chloroplasts under wounding stress. Thus, enhancement of the photosynthetic capacity in response to wounding is suggested to be crucial for stress adaptation in *P. patens*.

Additionally, the increased abundance of proteins related to photosystems leads to the production of reactive oxygen species (ROS), which can oxidize polyunsaturated lipids in plastid membranes. Hydroperoxidation of *a*-linolenic acid is required to increase the accumulation of OPDA [4]. Wound-induced proteins that are associated with photosystems may be involved in supplying oxygen for OPDA biosynthesis [4].

#### 2.5.6. Proteins Involved in Glycolysis, the TCA Cycle, and Energy Synthesis

**Figure 7:**
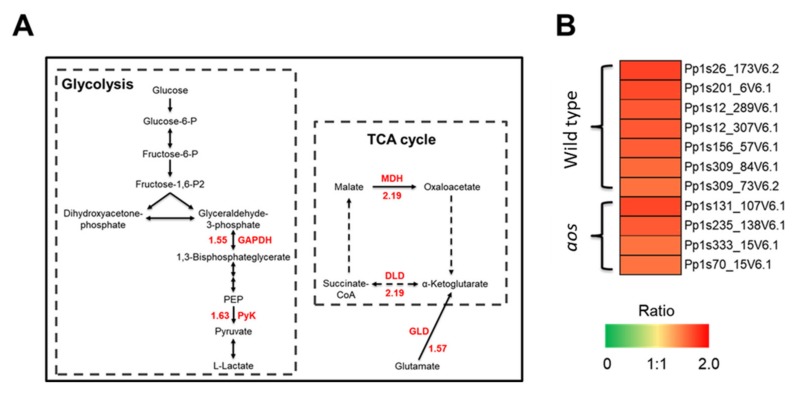
Differentially accumulated proteins involved in glycolysis and the TCA cycle. (**A**) Proteins involved in glycolysis and the TCA cycle were all up-regulated in response to wounding stress in wild-type *P. patens*. Red numbers represent the relative abundance of proteins related to glycolysis and the TCA cycle in response to wounding in wild-type. GAPDH (Pp1s309_84V6.1, Pp1s309_73V6.2), glyceraldehyde-3-phosphate dehydrogenase; PyK (Pp1s12_289V6.1, Pp1s12_307V6.1), pyruvate kinase; MDH (Pp1s38_300V6.1, Pp1s39_428V6.1, Pp1s79_110V6.1), malate dehydrogenase; DLD (Pp1s98_132V6.1), dihydrolipoamide dehydrogenase; GLD (Pp1s62_236V6.4), glutamate dehydrogenase. (**B**) Heat map representing the profile of differentially accumulated glycolysis and TCA cycle-related proteins induced by wounding in *P. patens*. Red indicates high accumulation, whereas green indicates low accumulation.

Wounding caused the accumulation of proteins involved in glycolysis, the TCA cycle, and energy synthesis in the wild-type plant (Figure 3 and Figure 7, Table S1); these proteins included pyruvate kinase, UDP-glucose pyrophosphorylase, malate dehydrogenase, phosphoenolpyruvate carboxykinase, pyruvate dehydrogenase E1 component subunit β, glyceraldehyde-3-phosphate dehydrogenase (GAPDH), ATPase, ATP synthase β chain, and vacuolar ATPase β subunit. The number of proteins involved in glycolysis, the TCA cycle, and energy synthesis was greater in the wild-type plant than in the *aos* mutant in response to wounding (Figure 3, Tables S1 and S2). Glycolysis and the TCA cycle are required for the efficient conversion of photosynthetic products to ATP. Proteins involved in energy synthesis, such as ATPase and ATP synthase, are related to the release of energy. Wounding appears to induce proteins involved in glycolysis and the TCA cycle to readily provide usable chemical energy.

The accumulation in photosystem-related proteins causes increased energy production, and effective energy release is needed for various physiological responses to stress [35]. The orchestrated accumulation of proteins involved in glycolysis, the TCA cycle, and energy synthesis, accompanied by the accumulation of photosystem-related proteins, is suggested to promote the effective utilization of light energy in *P. patens.*

#### 2.5.7. Proteins for Reactive Oxygen Scavenging

Wounding increased the abundance of superoxide dismutase in the wild-type plant but not in the *aos* mutant (Figure 3, Table S1). Superoxide dismutase catalyzes the dismutation of the superoxide radical (O_2_^−^) into oxygen (O_2_) or hydrogen peroxide (H_2_O_2_) [42]. Photosynthesis is enhanced by wounding but also produces ROS, which is likely harmful to cells. The accumulation of superoxide dismutase, which acts as an antioxidant, protects the cellular components from oxidation by ROS [42]. Therefore, wounding likely induces superoxide dismutase to reduce the accumulated ROS produced by photosynthesis to decrease oxidative stress.

### 2.6. Quantitative RT-PCR Analysis of Genes Encoding Proteins Accumulated by Wounding

**Figure 8:**
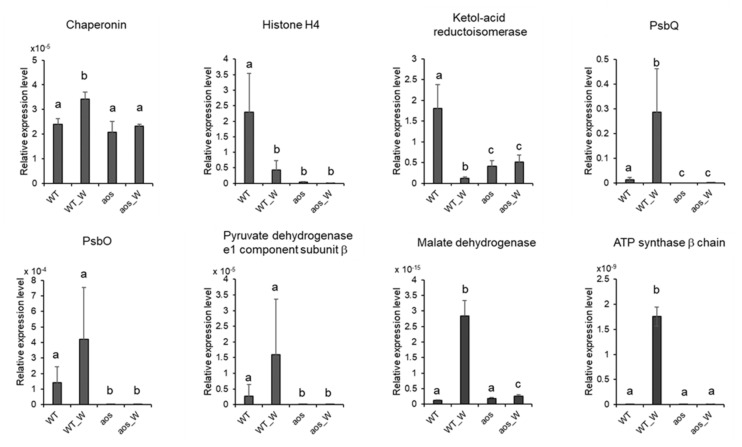
qRT-PCR analyses of genes encoding wound-accumulative proteins. The transcription levels of selected genes belonging to different groups were analyzed. The selected genes included the following: Chaperonin (Pp1s141_125V6.1); Histone H4 (Pp1s342_32V6.1); Ketol-acid reductoisomerase (Pp1s60_179V6.1); Photosystem II oxygen evolving complex Protein PsbQ (Pp1s182_26V6.1); Photosystem II manganese-stabilizing protein PsbO (Pp1s306_84V6.1); Pyruvate dehydrogenase e1 component subunit β (Pp1s156_57V6.1); Malate dehydrogenase (Pp1s79_110V6.1) and ATP synthase β chain (Pp1s310_30V6.1). WT, wild-type without treatment; WT_W, wild-type treated with wounding for 3 h; aos, *aos* mutant without treatment; aos_W, *aos* mutant treated with wounding for 3 h. The values are the mean ± SD (*n* = 3). Relative gene expression levels were normalized to actin. The data are shown as the mean ± SD from three independent biological replicates. Different letters indicate that the change is significant as determined by one-way ANOVA according to Tukey’s multiple comparison test (*p* < 0.05).

To investigate the correlation between protein accumulation and gene expression, we selected eight genes encoding proteins, which were accumulated in the wild-type plant subjected to wounding and performed qRT-PCR analyses of the genes (Figure 8). In eukaryotes, transcription occurs in the nucleus, and translation or protein synthesis take place in the cytoplasm. These processes are separated sequentially and spatially. Moreover, proteins are modified by each organelle such as phosphorylation and glycosylation, and then many of proteins become mature. In most cases, genes are expressed before accumulation of proteins. These processes are required for minutes to hours. Therefore, samples were collected at 3 h after wounding in this study. The results showed that wounding induced the transcriptional levels of four genes encoding proteins, chaperonin, PsbQ, malate dehydrogenase, and ATP synthase β chain, in the wild-type plant. In the case of these proteins, wound-induced gene expression was in accordance with wound-induced accumulation of proteins. In contrast, the expression of four other genes encoding proteins, histone H4, ketol-acid reductoisomerase, PsbO, and pyruvate dehydrogenase e1 component subunit β, was not provoked by wounding in the wild-type plant. These data indicated that protein accumulation did not always coincide with gene expression in wounded *P. patens*. When *P. patens* was subjected to wounding, the degradation of histone H4, ketol-acid reductoisomerase, PsbO, and pyruvate dehydrogenase e1 component subunit β was possibly suppressed.

### 2.7. Comparison of Proteomic Data in This Study with Those in P. Patens Treated with OPDA

OPDA treatment and wounding increased the levels of many proteins related to photosystems in the wild-type plant (Figure 3, Table S1) [30]; however, the abundance of photosystem-related proteins was not increased by wounding in the *aos* mutant (Figure 3, Table S2). OPDA treatment has previously been shown to increase the abundance of proteins related to photosynthesis in wild-type *P. patens* [30,41]. These data suggested that wounding induces proteins involved in photosynthesis through OPDA signaling in *P. patens.*

**Figure 9:**
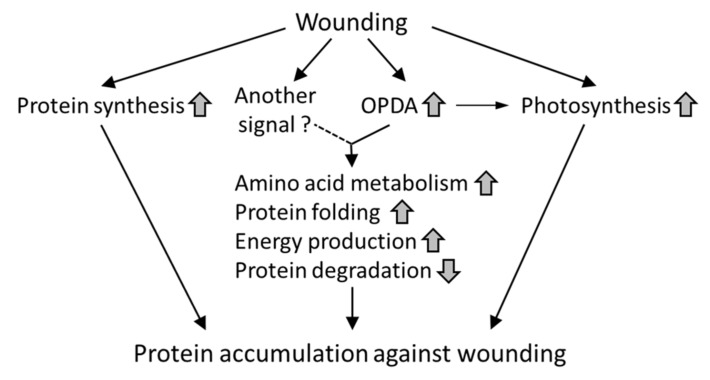
Proposed model for protein accumulation in response to wounding in *P. patens.* Upward or downward arrows indicate an increase or decrease, respectively.

In contrast to wounded *P. patens*, proteins involved in protein synthesis and protein folding were decreased in OPDA-treated *P. patens* [30]. Wounding stimulates a wide variety of signaling systems. OPDA-mediated signaling is one of wound-induced physiological responses in *P. patens*. Various types of signaling triggered by wounding are probably involved in the crosstalk with OPDA signaling in *P. patens* (Figure 9). Therefore, the proteins accumulated in wounded *P. patens* do not completely correspond to the proteins whose abundance is altered in *P. patens* treated with OPDA. 

In a previous study, *P. patens* was subjected to 10 μM OPDA for 24 h [30]. However, the OPDA concentration was transiently increased by wounding until 2 h after wounding and decreased to basal levels after 4 h. in *P. patens* (Figure 1). It appears that *P. patens* subjected to wounding was not significantly affected by OPDA at 4 h after wounding. The difference of the period under the influence of OPDA may cause differential accumulation of proteins between wounded *P. patens* and OPDA-treated *P. patens*.

## 3. Materials and Methods

### 3.1. Plant Growth Conditions and Treatment

The moss *Physcomitrella patens* Gransden 2004 strain was used as wild-type *P. patens* and was grown on 20 mL of BCDAT agar medium [43] for 3 weeks in a 9-cm Petri dish under continuous white fluorescent light (40 μmol photons m^−2^s^−1^) at 25°C. To generate wounding stress, whole gametophores were wounded with tweezers.

### 3.2. Analysis of OPDA Concentration in P. Patens

Gametophores of *P. patens* (approximately 200 mg), which were grown on BCDAT agar medium for 3 weeks, were frozen in liquid nitrogen and extracted with 10 mL of ethanol. The concentration of OPDA was analyzed by UPLC-MS/MS according to the method of Sato et al [44]. Four independent experiments were conducted as biological replicates. One-way analysis of variance (ANOVA) followed by Tukey’s multiple comparison was used for comparison among multiple groups and conducted using R (version 3.6.0). A *p*-value of < 0.05 was considered as statistically significant.

### 3.3. Generation of a P. Patens Mutant with Disrupted PpAOS1 and PpAOS2

To construct a vector for *PpAOS1* gene disruption, a 1.0-kb genomic DNA fragment beginning 5′ to *PpAOS1* was amplified using KOD FX DNA polymerase (Toyobo, Osaka, Japan), and the primers PpAOS1KO5′-F and PpAOS1KO5′-R. A 1.0-kb genomic DNA fragment ending 3′ to *PpAOS1* was also amplified using primers PpAOS1KO3′-F and PpAOS1KO3′-R. Each fragment was cloned into the vector pBluescript SKII (+) (Merck, Darmstadt, Germany). The 5′ *PpAOS1* genomic fragment was digested with *Xba*I (Takara Bio Inc., Shiga, Japan) and *EcoR*I (Takara Bio Inc., Shiga, Japan) and inserted into pTN182, which carries a G418-resistant cassette, digested with *Xba*I (Takara Bio Inc., Shiga, Japan) and *EcoR*I (Takara Bio Inc., Shiga, Japan) to obtain pTN182-PpAOS1KO5′. Similarly, the 3′ *PpAOS1* genomic fragment was inserted into pTN182-PpAOS1KO5′, which had been digested with *Sph*I (Takara Bio Inc., Shiga, Japan) and *Nde*I, (Takara Bio Inc., Shiga, Japan) to yield pTN182-PpAOS1KO.

A vector for *PpAOS2* gene disruption was constructed using pTN186-PpAOS2KO. A 1.0-kb genomic DNA fragment beginning 5′ to *PpAOS2* was amplified using KOD FX DNA polymerase (Toyobo, Osaka, Japan) and primers PpAOS2KO5′-F and PpAOS2KO5′-R. A 1.0-kb genomic DNA fragment ending 3′ to *PpAOS2* was also amplified using primers PpAOS2KO3′-F and PpAOS2KO3′-R. Each fragment was cloned into pBluescript SKII (+) (Merck, Darmstadt, Germany). The 5′ *PpAOS2* genomic fragment was digested with *Kpn*I (Takara Bio Inc., Shiga, Japan) and *HindIII* (Takara Bio Inc., Shiga, Japan) and inserted into pTN186, which contained a hygromycin-resistance cassette and had been digested with *Kpn*I (Takara Bio Inc., Shiga, Japan) and *HindIII* (Takara Bio Inc., Shiga, Japan) to obtain pTN186-PpAOS2KO5′. The 3′ *PpAOS2* genomic fragment was inserted into pTN186-PpAOS2KO5′, which had been digested with *Sph*I (Takara Bio Inc., Shiga, Japan) and *Sac*I (Takara Bio Inc., Shiga, Japan), to yield pTN186-PpAOS2KO. 

The plasmids pTN182-PpAOS1KO and pTN186-PpAOS2KO contained *Bam*HI and *Kpn*I restriction sites, respectively. Polyethylene glycol-mediated transformation was conducted as reported previously by Nishiyama et al [43]. The selected plants were incubated for an additional week without antibiotics and then transferred again onto selection medium. Stable transformants were chosen by PCR using appropriate primer sets (5′ end of *PpAOS1*: PpAOS2KO5′-F2 and Pcmv-R; 3′ end of *PpAOS1*: 35SPS-F and PpAOS2KO3′-R2; 5′ end of *PpAOS2*: PpAOS1KO5′-F2 and Pcmv-R; 3′ end of *PpAOS2*: 35SPS-F and PpAOS1KO3′-R2) to confirm the integration of the selectable marker into the targeted genes *PpAOS1* and *PpAOS2*. The primers used in this experiment are listed in Supplementary Table S3.

### 3.4. Protein Extraction

*P. patens* gametophores were grown for 3 weeks and then wounded with tweezers. At 24 h after wounding, approximately 500 mg of *P. patens* fresh tissue was ground into powder in liquid nitrogen using a mortar and pestle. The powder was transferred into a solution of 10% trichloroacetic acid and 0.07% 2-mercaptoethanol in acetone and mixed. The suspension was sonicated for 5 min and then incubated for 45 min at −20 °C. After this incubation, the suspension was centrifuged at 9, 000 × *g* for 20 min at 4 °C. The resulting supernatant was discarded, and the pellet was washed three times with 3 mL of acetone containing 0.07% 2-mercaptoethanol. The final pellet was dried using a vacuum pump. The pellet was resuspended by vortexing for 1 h at 25 °C in 5 mL of lysis buffer consisting of 100 mM Tris-HCl (pH 8.5), 2% SDS, and 50 mM dithiothreitol (DTT). The suspension was then centrifuged at 20,000 × *g* for 20 min at 25 °C. The resulting supernatant was collected as the total protein solution. Three independent experiments were conducted as biological replicates. The concentration of the protein solution was measured using the Lowry method [45].

### 3.5. Digestion of Proteins

For in-solution digestion, 100 µg of protein was subjected to chloroform/methanol extraction [46]. The pellet was resuspended with 50 mM NH_4_HCO_3_. The solution was reduced with 50 mM DTT and then alkylated with 50 mM iodoacetamide. Proteins were digested using trypsin and lysyl endopeptidase at a 1:100 enzyme/protein ratio at 37 °C for 16 h [33].

### 3.6. Nanoliquid Chromatography-Tandem MS Analysis

Peptide separation and detection were performed using an Ultimate 3000 nano LC (Thermo Fisher Scientific, San Jose, CA, USA) and an LTQ Orbitrap mass spectrometer (Thermo Fisher Scientific, San Jose, CA, USA). The system was operated in data-dependent acquisition mode with XCalibur software (ver. 2.0.7, Thermo Fisher Scientific, San Jose, CA, USA). The peptides were loaded onto a C18 PepMap trap column (300 µm ID × 5 mm, Dionex, Thermo Fisher Scientific, San Jose, CA, USA). The peptides were eluted with a linear acetonitrile gradient (8–30% over 150 min) in 0.1% formic acid in acetonitrile at a flow rate of 200 nL/min and were loaded and separated on a C18 capillary tip column (75 µm ID × 120 mm, nano LC capillary column, NTTC-360/75-3, Nikkyo Technos, Tokyo, Japan) with a spray voltage of 1.5 kV. Elution was performed with a linear acetonitrile gradient (5-25% in 120 min) in 0.1% formic acid. Full-scan mass spectra were acquired in the Orbitrap over 400-1,500 *m/z* with a resolution of 30,000. A lock mass function was used to obtain high mass accuracy [47]. The top ten most intense precursor ions were selected for collision-induced fragmentation in the linear ion trap at a normalized collision energy of 35%. Dynamic exclusion was employed within 90 s to prevent the repetitive selection of the peptides [48].

### 3.7. Protein Identification Using Mascot

Proteins were identified from the acquired MS/MS spectra using Mascot software (ver. 2.5.1, Matrix Science, London, UK) and from the *P. patens* database (38,480 protein sequences) and a contaminant database (262 protein sequences) with Proteome Discoverer (ver. 1.4.0.288, Thermo Fisher Scientific, San Jose, CA, USA). The *P. patens* database was obtained from the Phytozome database (ver. 11. 0. 9, http://www.phytozome.net/). The parameters used in the Mascot searches were as follows: the carbamidomethylation of cysteine was set as a fixed modification; the oxidation of methionine was set as a variable modification; trypsin was specified as the proteolytic enzyme; and one missed cleavage was allowed. The peptide mass tolerance was set at 10 ppm. The fragment mass tolerance was set at 0.8 Da, and the peptide charge was set at +2, +3, and +4. An automatic decoy database search was performed within the search. The Mascot results were filtered using the percolator function in Proteome Discoverer to improve the accuracy and sensitivity of peptide identification [49]. False discovery rates for the identification of all searches were less than 1.0%. Peptides with a percolator ion score of more than 13 (student’s *t*-test, *p* < 0.05) were used for protein identification. 

### 3.8. Analysis of Differentially Accumulated Proteins using the Acquired MS Data

For differential analyses, the commercial label-free quantification package SIEVE (ver. 2.1, Thermo Fisher Scientific, San Jose, CA, USA) was used to compare the relative abundance of peptides and proteins between the control and experimental groups. The chromatographic peaks detected by MS were aligned, and the peptide peaks were detected as frames using the following settings: the frame time width was 5.0 min; the frame *m/z* width was 10 ppm, and frames were produced on all parent ions subjected to MS/MS scanning. The frames with MS/MS scans were matched to the imported Mascot results. In the analysis of differential protein abundance, the total ion current was used for normalization. The minimum requirement for the identification of a protein was two matched peptides and a *p* value of < 0.05.

### 3.9. Classification of Proteins and Bioinformatic Analysis

The functions of the identified proteins were categorized according to the annotations of the cosmoss.org *P. patens* database (http://www.cosmoss.org/) and the EU *Arabidopsis thaliana* genome project [50]. The functional interactions of the identified proteins were examined using STRING (version 10.5, http://string-db.org/) [51]. Briefly, the protein list was subjected to Blast searches against the *Physcomitrella patens* STRING database, which includes the physical and functional relationships of protein molecules, supported by associations derived from eight lines of evidence: the neighborhood in the genome; gene fusions; cooccurrence across the genome; coexpression; experimental/biochemical data; information in databases (associations in curated databases); text-mining (comentioned in PubMed abstracts); and homology [52]. The biological pathways of the identified proteins were deduced from KEGG analysis (http://www.genome.jp/kegg/). The results of GO functional and pathway enrichment analysis and the pathway enrichment of the identified proteins were analyzed using an international standardized gene functional classification system (http://www.geneontology.org/), employing settings in reference to previous reports [33,53]. Protein subcellular localization was predicted using TargetP (http://www.cbs.dtu.dk/services/TargetP/), Bacello (http://gpcr2.biocomp.unibo.it/bacello/index.htm) and WoLF PSORT (http://wolfspsort.org/) [54]. Multiple sequence alignments were conducted by using Clustal Omega (http://www.ebi.ac.uk/Tools/msa/clustalo/).

### 3.10. Quantitative RT-PCR

The coding sequences (CDSs) for the selected genes were used to design specific primers for quantitative RT-PCR (qRT-PCR). *P. patens* total RNA was extracted using the Isospin Plant RNA kit (Nippon gene, Tokyo, Japan) according to the manufacturer’s instructions. First-strand cDNA prepared by M-MLV reverse transcriptase (Invitrogen, Carlsbad, CA, USA) was used as the template. To analyze gene expression levels of a chaperonin gene (Pp1s141_125V6.1), the KOD SYBR qPCR Mix (Toyobo, Osaka, Japan) was used according to the manufacturer’s protocol. Each reaction mixture contained 12.5 µL of KOD SYBR qPCR Mix, 1 µL of each primer (10 mM), 1 µL of cDNA, and 9.5 µL of MilliQ water. qRT-PCR was performed on a Thermal Cycler Dice Real-Time system (TP800, Takara Bio Inc., Shiga, Japan). To analyze expression levels of other genes, the TB Green^TM^ Premix Ex Taq^TM^ II (Takara Bio Inc., Shiga, Japan) was used according to the manufacturer’s protocol. Each reaction mixture contained 10 µL of TB Green *Premix Ex Taq* II (Takara Bio Inc., Shiga, Japan), 0.4 µL of each primer (10 mM), 2 µL of cDNA, 0.4 µL ROX Reference Dye, and 6.8 µL of MilliQ water. qRT-PCR was performed on a Thermal Cycler Dice Real-Time system (Applied Biosystem 7000, Thermo Fisher Scientific, United Kingdom). The qRT-PCR conditions were as follows: preincubation at 95°C for 30 s followed by 40 cycles of 95°C for 5 s and 60°C for 30 s. The specificity of each PCR amplicon was assessed with a dissociation curve (95°C for 15 s, 60°C for 30 s, and 95°C at 15 s). Actin (accession no: AW698983) was used as an internal standard for the normalization of gene expression, and the actin level was set to 1.0. The primers used in this experiment are listed in Supplementary Table S4.

## 4. Conclusions

This study revealed that wounding mainly promoted the accumulation of proteins involved in protein synthesis, amino acid synthesis, photosynthesis, protein folding, and glycolysis. Because these wounding-responsive proteins are also found in flowering plants, the accumulation of these proteins in response to wounding may be conserved in land plants. The comparison of proteomic data from the wild-type and the *aos* mutant suggests that *PpAOS* gene expression, which leads to an increase in OPDA, enhances photosynthesis and effective energy utilization in response to wounding in *P. patens.* The present data will help our understanding of adaptive signaling in response to wounding in land plants, as well as the effects of *AOS* gene expression for stress adaptation in *P. patens.*

## Supplementary Materials

Supplementary materials can be found at https://www.mdpi.com/1422-0067/21/4/1417/s1.

## Abbreviations

AOS	Allene oxide synthase
COI1	Coronatine insensitive 1
12,13-EOT	12,13(*S*)-epoxyoctadecatrienoic acid
GAPDH	Glyceraldehyde-3-phosphate dehydrogenase
GLD	Glutamate dehydrogenase
GO	Gene Ontology
HPL	Hydroperoxide lyase
13-HPOT	13(*S*)-hydroperoxyoctadecatrienoic acid
HSP	Heat shock protein
JA	Jasmonic acid
JA-Ile	Isoleucine conjugate of jasmonic acid
KEGG	Kyoto Encyclopedia of Genes and Genomes
LC-MS/MS	Liquid chromatography-tandem mass spectrometry
LEA	Late embryogenesis abundant
MDH	Malate dehydrogenase;
MeJA	Methyl jasmonate
OPDA	12-Oxo-phytodienoic acid
qRT-PCR	Quantitative real-time polymerase chain reaction
ROS	Reactive oxygen species
Rubisco	Ribulose-1,5-bisphosphate carboxylase/oxygenase
STRING	Search Tool or the Retrieval of Interacting Genes
UPLC-MS/MS	Ultra-performance liquid chromatography-tandem mass spectrometry
