# Supplementary material for: Comparative Proteomic Analysis of Wild-Type Physcomitrella Patens and an OPDA-Deficient Physcomitrella Patens Mutant with Disrupted PpAOS1 and PpAOS2 Genes after Wounding
Source: Int J Mol Sci. 2020 Feb 19;21(4):1417. doi: 10.3390/ijms21041417 (PMC7073133; doi:10.3390/ijms21041417)
Supplement: Supplementary file 1 [file ijms-21-01417-s001.pdf]

Supplementary Material

Title

Comparative proteomic analysis of wild-type *Physcomitrella patens* and an OPDA-deficient *Physcomitrella patens* mutant with disrupted *PpAOS1* and *PpAOS2* genes after wounding

Authors

Weifeng Luo[a], Setsuko Komatsu[b], Tatsuya Abe[a], Hideyuki Matsuura[a], and Kosaku Takahashi[a,c*]

Affiliations

[a]Research Faculty of Agriculture, Hokkaido University, Sapporo 606-8589, Japan

[b]Department of Environmental and Food Sciences, Faculty of Environmental and Information Sciences,
Fukui University of Technology, 3-6-1 Gakuen, Fukui 910-8505, Japan

[c]Department of Nutritional Science, Faculty of Applied BioScience, Tokyo University of Agriculture, Tokyo 165-8502, Japan.

*To whom correspondence should be addressed: Tel.: +81-3-5477-2679; E-mail: kt207119@nodai.ac.

Supplementary Fig. S1.

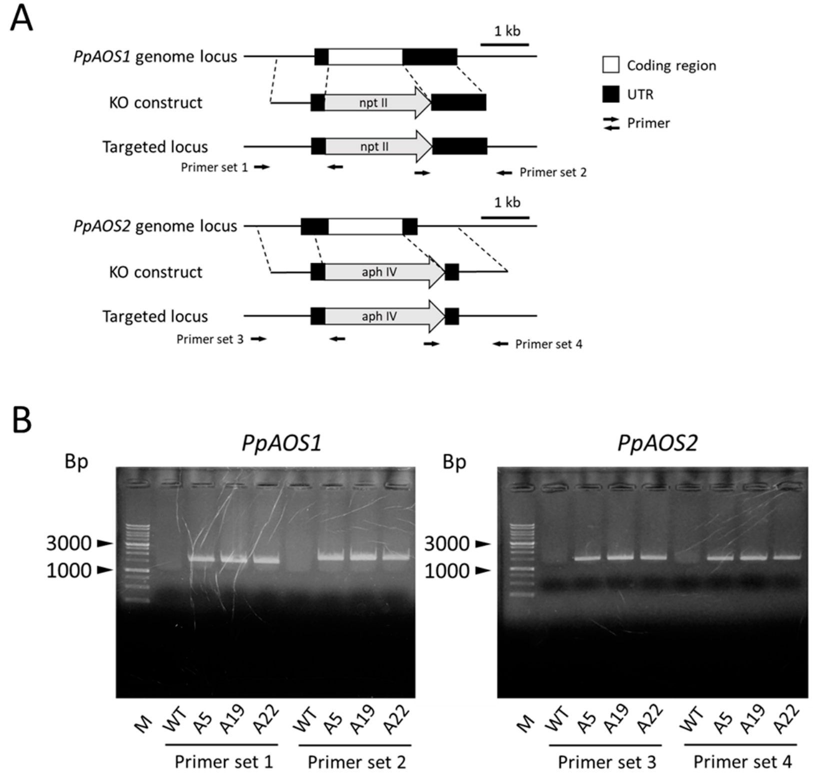

Fig. S1. Disruption of *PpAOS1* and *PpAOS2* genes in *P. patens.*

A, Genomic structures of *PpAOS1* and *PpAOS2* in the wild-type and targeted *PpAOS1* and *PpAOS2* knock-out mutants (A5, A19, and A22). The npt II and aph IV expression cassettes were inserted in A5, A19, and A22 strains. B, Genomic PCR data of wild-type, and A5, A19 and A22 strains. *P. patens* genomic DNA was isolated from protonemata by the CTAB method (Nishiyama et al., Ref. 32). PCR was performed in 50 µL of a reaction mixture containing 1 µL of genomic DNA solution, 1.5 µL of each primer (5 µM), 25 µL of KOD One PCR Master Mix (Toyobo, Japan), and 21 µL of Milli-Q water. PCR was conducted with the following conditions: 30 cycles of 98°C for 10 s, 58°C for 5 s, and 68°C for 15 s. The PCR products were analyzed by gel electrophoresis and visualized by ethidium bromide. The primers used are listed in Supplemental Table

S5.

Supplementary Fig. S2.

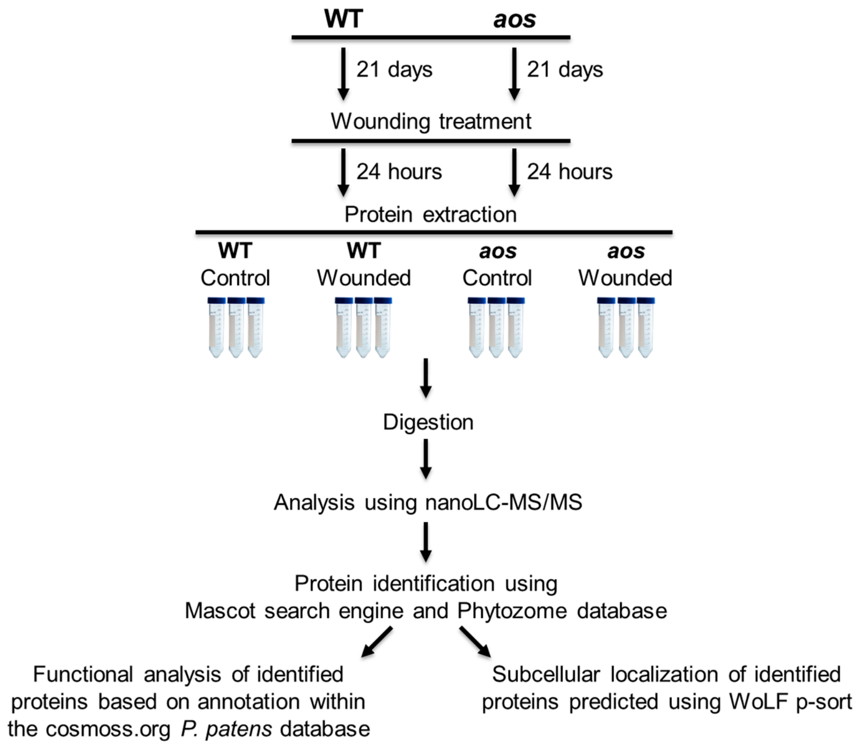

Fig. S2. Experimental design for the proteomic analysis of *P. patens* proteins.

Three-week-old *P. patens* (wild-type and the *aos* mutant) were treated with wounding treatment. *P. patens* (wild-type and the *aos* mutant) without wounding treatment were used as the controls. Extracted proteins were digested, and the obtained peptides were analyzed using nano LC-MS/MS. Three independent experiments were conducted as biological replicates for the proteome experiments.

Supplementary Table S1. Proteins identified as responsive to wounding in wild-type *P. patens*.

Function	Protein ID[1]	Description	Subcellular location	MP[2]	Ratio[3]
Protein synthesis	Pp1s207_94V6.1	Protein synthesis factor, GTP-binding	Endoplasmic reticulum	3	2.78
Protein synthesis	Pp1s73_232V6.2	Ribosomal Protein L14b/L23e	Endoplasmic reticulum	4	2.73
Protein synthesis	Pp1s114_79V6.1	Ribosomal Protein L14b/L23e	Endoplasmic reticulum	4	2.66
Protein synthesis	Pp1s16_112V6.1	Ribosomal Protein L14b/L23e	Endoplasmic reticulum	4	2.66
Protein synthesis	Pp1s62_136V6.1	Ribosomal Protein L14b/L23e	Endoplasmic reticulum	2	2.66
Protein synthesis	Pp1s330_36V6.1	Ribosomal Protein L3	Cytoplasm	3	2.12
Protein synthesis	Pp1s21_165V6.1	Ribosomal Protein 60S	Cytoplasm	3	2.02
Protein synthesis	Pp1s14_438V6.1	Ribosomal Protein S13	Endoplasmic reticulum	4	2.02
Protein synthesis	Pp1s21_36V6.1	DJ-1/PfpI family	Endoplasmic reticulum	2	2.01
Protein synthesis	Pp1s144_37V6.1	Ribosomal Protein L19	Endoplasmic reticulum	3	2.01
Protein synthesis	Pp1s145_172V6.1	Elongation factor 1-delta 1	Endoplasmic reticulum	3	2.00
Protein synthesis	Pp1s233_94V6.1	Rnase l inhibitor-like Protein	Endoplasmic reticulum	7	2.00
Protein synthesis	Pp1s402_8V6.1	Rnase l inhibitor-like Protein	Endoplasmic reticulum	3	2.00
Protein synthesis	Pp1s10_102V6.1	Ribosomal Protein L4/L1e	Endoplasmic reticulum	4	1.91
Protein synthesis	Pp1s302_25V6.1	Ribosomal Protein L19/L19e	Endoplasmic reticulum	6	1.79
Protein synthesis	Pp1s3_375V6.1	Ribosomal Protein L19/L19e	Endoplasmic reticulum	4	1.79
Protein synthesis	Pp1s83_173V6.1	Ribosomal Protein L19/L19e	Endoplasmic reticulum	2	1.76
Protein synthesis	Pp1s73_232V6.2	Ribosomal Protein L14b/L23e	Endoplasmic reticulum	2	1.75
Protein synthesis	Pp1s50_102V6.1	Ribosomal Protein L19/L19e	Cytoplasm	10	1.70
Protein synthesis	Pp1s17_304V6.2	Ribosomal Protein S27e	Endoplasmic reticulum	11	1.65
Protein synthesis	Pp1s240_91V6.1	Serine threonine-Protein kinase	Cytoplasm	16	1.63
Protein synthesis	Pp1s215_71V6.1	Ribosomal Protein S14	Cytoplasm	2	1.59
Protein synthesis	Pp1s72_222V6.1	Ribosomal Protein S15	Cytoplasm	2	1.59

Continued

Supplementary Table S1. Continued.

Function	Protein ID[1]	Description	Subcellular location	MP[2]	Ratio[3]
Protein synthesis	Pp1s379_40V6.1	Signal transduction Protein with cbs domains	Endoplasmic reticulum	9	1.51
Amino acid metabolism	Pp1s60_179V6.1	Ketol-acid reductoisomerase	Chloroplast	3	2.37
Amino acid metabolism	Pp1s290_40V6.1	Dehydrogenase, multihelical	Cytoplasm	2	2.37
Amino acid metabolism	Pp1s228_3V6.1	Acetohydroxy acid isomeroreductase	Chloroplast	6	2.12
Amino acid metabolism	Pp1s40_57V6.2	Dihydropyrimidine dehydrogenase	Chloroplast	9	1.64
Amino acid metabolism	Pp1s17_59V6.1	Pyridoxal phosphate-dependent enzyme (oas-tl4 cysteine)synthase	Mitochondrial	11	1.62
Amino acid metabolism	Pp1s27_81V6.1	Vitamin-b12 independent methionine 5-methyltetrahydropteroyltriglutamate-homocysteine	Chloroplast	9	1.62
Amino acid metabolism	Pp1s62_236V6.4	Glutamate dehydrogenase	Mitochondrial	6	1.57
Amino acid metabolism	Pp1s62_236V6.7	Glutamate dehydrogenase	Mitochondrial	4	1.56
Amino acid metabolism	Pp1s33_110V6.2	Vitamin-b12 independent methionine 5-methyltetrahydropteroyltriglutamate-homocysteine	Chloroplast	16	1.55
Amino acid metabolism	Pp1s99_201V6.1	Amino acid binding	Cytoplasm	4	1.51
Amino acid metabolism	Pp1s399_19V6.1	Vitamin-b12 independent methionine 5-methyltetrahydropteroyltriglutamate-homocysteine	Chloroplast	14	1.50
Amino acid metabolism	Pp1s131_72V6.1	Serine carboxypeptidase	Chloroplast	2	0.49
Protein fold	Pp1s141_125V6.1	Chaperonin (Cpn60/TCP-1)	Chloroplast	2	3.13
Protein fold	Pp1s201_109V6.1	Chaperonin (Cpn60/TCP-1)	Chloroplast	2	3.13
Protein fold	Pp1s56_219V6.1	Chaperonin (Cpn60/TCP-1)	Chloroplast	6	3.13
Photosystem	Pp1s359_40V6.1	Pyridoxal phosphate-dependent enzyme	Chloroplast	7	2.29
Photosystem	Pp1s78_56V6.2	Dihydrolipoamide acetyltransferase, long form	Chloroplast	7	2.26
Photosystem	Pp1s206_126V6.1	Germin-like Protein GLP2	Chloroplast	2	2.05
Photosystem	Pp1s112_169V6.1	Cytochrome b6-f complex iron-sulfur subunit	Chloroplast	3	2.01
Photosystem	Pp1s425_20V6.1	Geranylgeranyl reductase	Chloroplast	5	1.62

Continued

Supplementary Table S1. Continued.

Function	Protein ID[1]	Description	Subcellular location	MP[2]	Ratio[3]
Photosystem	Pp1s131_154V6.1	FerRedoxin--nadp+ reductase-like Protein	Chloroplast	2	1.59
Photosystem	Pp1s425_20V6.2	Geranylgeranyl reductase	Chloroplast	7	1.58
Photosystem	Pp1s20_284V6.1	Geranylgeranyl reductase	Chloroplast	5	1.57
Photosystem	NC_005087.1_cds id_NP_904171.1	Cytochrome b6	Chloroplast	3	1.53
Photosystem	Pp1s100_107V6.1	Geranylgeranyl reductase	Chloroplast	5	1.50
Photosystem I	Pp1s345_25V6.1	Photosystem I reaction centre Protein PsaF, subunit III	Chloroplast	5	2.20
Photosystem I	Pp1s80_23V6.1	Photosystem I reaction centre Protein PsaF, subunit III	Chloroplast	5	2.19
Photosystem I	Pp1s121_54V6.1	Photosystem I reaction centre Protein PsaF, subunit III	Chloroplast	5	2.13
Photosystem I	Pp1s19_276V6.1	Photosystem I reaction centre Protein PsaF, subunit III	Chloroplast	5	2.13
Photosystem I	Pp1s334_17V6.1	Photosystem I reaction centre subunit IV/PsaE	Chloroplast	4	0.66
Photosystem I	NC_005087.1_cds id_NP_904203.1	Photosystem I P700 chlorophyll a apoprotein A2	Chloroplast	7	0.50
Photosystem II	Pp1s25_66V6.1	Photosystem II manganese-stabilizing Protein PsbO	Chloroplast	8	2.13
Photosystem II	Pp1s182_26V6.1	Photosystem II oxygen evolving complex Protein PsbQ	Chloroplast	3	1.86
Photosystem II	Pp1s306_84V6.1	Photosystem II manganese-stabilizing Protein PsbO	Chloroplast	6	1.84
Photosystem II	Pp1s60_65V6.1	Photosystem II manganese-stabilizing Protein PsbO	Chloroplast	2	1.65
Photosystem II	Pp1s628_7V6.1	Light-harvesting complex ii Protein lhcb5	Chloroplast	3	1.61
Photosystem II	Pp1s6_313V6.1	Light-harvesting complex ii Protein lhcb5	Chloroplast	11	1.60
Photosystem II	Pp1s254_25V6.1	Chloroplast precursor (Plastocyanin)	Chloroplast	5	1.52
Photosystem II	Pp1s214_86V6.1	Type iii chlorophyll a b-binding Protein	Chloroplast	3	1.50
Photosystem II	Pp1s214_87V6.1	Type iii chlorophyll a b-binding Protein	Chloroplast	3	1.50
Photosystem II	Pp1s429_33V6.1	Type iii chlorophyll a b-binding Protein	Chloroplast	3	1.50
Photosystem II	Pp1s259_76V6.1	Photosystem II 5 kDa Protein, chloroplast precursor (PSII-T)	Chloroplast	2	0.51
Photosystem II	Pp1s54_166V6.1	Photosystem II Protein PsbR (Photosystem ii 10 kda polypeptide)	Chloroplast	3	0.50

Continued

Supplementary Table S1. Continued.

Function	Protein ID[1]	Description	Subcellular location	MP[2]	Ratio[3]
Glycolysis	Pp1s26_173V6.2	QuinohaemoProtein ethanol dehydrogenase type I (QH-EDH1)	Plasma Membrane	15	1.72
Glycolysis	Pp1s201_6V6.1	Mitochondrial nad-dependent malate dehydrogenase	Cytoplasm	2	1.69
Glycolysis	Pp1s12_289V6.1	Pyruvate kinase	Cytoplasm	5	1.63
Glycolysis	Pp1s12_307V6.1	Pyruvate kinase	Cytoplasm	6	1.63
Glycolysis	Pp1s156_57V6.1	Pyruvate dehydrogenase e1 component subunit beta	Cytoplasm	12	1.61
Glycolysis	Pp1s309_84V6.1	Glyceraldehyde-3-phosphate dehydrogenase (GAPDH)	Cytoplasm	9	1.55
Glycolysis	Pp1s309_73V6.2	Glyceraldehyde-3-phosphate dehydrogenase (GAPDH)	Cytoplasm	9	1.52
Energy synthesis	Pp1s103_66V6.1	ATPase	Chloroplast	3	2.32
Energy synthesis	Pp1s294_50V6.1	Dihydroorotate dehydrogenase family Protein	Mitochondrial	2	1.64
Energy synthesis	Pp1s154_66V6.1	ATP synthase beta chain	Mitochondrial	5	1.63
Energy synthesis	Pp1s154_69V6.1	ATPase alpha subunit Protein ATPB	Mitochondrial	17	1.58
Energy synthesis	Pp1s310_30V6.1	ATP synthase beta chain	Mitochondrial	17	1.58
Energy synthesis	Pp1s100_117V6.1	ATP synthase subunit beta	Mitochondrial	6	1.52
Energy synthesis	Pp1s425_12V6.1	Vacuolar ATPase b subunit	Mitochondrial	6	1.52
Energy synthesis	Pp1s85_75V6.2	ATP synthase subunit beta	Mitochondrial	6	1.52
Energy synthesis	Pp1s15_183V6.1	AMP-ACTIVATED Protein KINASE	Chloroplast	2	0.35
TCA cycle	Pp1s38_300V6.1	Malate dehydrogenase	Cytoplasm	7	2.19
TCA cycle	Pp1s98_132V6.1	Dihydrolipoamide dehydrogenase	Mitochondrial	3	2.16
TCA cycle	Pp1s39_428V6.1	Malate dehydrogenase	Cytoplasm	8	2.15
TCA cycle	Pp1s79_110V6.1	Malate dehydrogenase	Cytoplasm	9	1.70
Redox	Pp1s131_71V6.3	Superoxide dismutase	Chloroplast	2	2.31
Redox	Pp1s40_48V6.3	Multicopper oxidase type 2	Cytoplasm	6	1.91
Redox	Pp1s308_11V6.1	L-ascorbate oxidase	Cytoplasm	3	1.89
Redox	Pp1s106_16V6.2	Formate dehydrogenase	Cytoplasm	4	1.87
Redox	Pp1s131_153V6.1	Superoxide dismutase (SOD)	Chloroplast	11	1.85

Continued

Supplementary Table S1. Continued.

Function	Protein ID[1]	Description	Subcellular location	MP[2]	Ratio[3]
Redox	Pp1s106_68V6.2	Thioredoxin m	Mitochondrial	5	1.62
Redox	Pp1s23_109V6.1	Thioredoxin m	Mitochondrial	5	1.62
Redox	Pp1s317_49V6.1	Thioredoxin m	Mitochondrial	5	1.62
Redox	Pp1s326_66V6.1	Thioredoxin m	Mitochondrial	9	1.62
Carbohydrate metabolism	Pp1s98_250V6.1	GDP-mannose 3 -epimerase	Cytoplasm	2	2.01
Carbohydrate metabolism	Pp1s283_22V6.1	UDP-glucose pyrophosphorylase	Cytoplasm	3	1.92
Carbohydrate metabolism	Pp1s39_82V6.1	UDP-glucose pyrophosphorylase	Cytoplasm	10	1.71
Carbon fixation	Pp1s251_44V6.1	Ribulose bisphosphate carboxylase, small chai(RuBisCO)	Chloroplast	4	2.19
Carbon fixation	Pp1s114_95V6.1	Rubisco subunit binding-Protein alpha subunit	Chloroplast	6	0.56
Carbon fixation	Pp1s170_46V6.1	Rubisco subunit binding-Protein alpha subunit	Chloroplast	2	0.48
Gene expression	Pp1s133_103V6.1	Histone h4	Nuclear	3	1.99
Gene expression	Pp1s269_48V6.1	Histone h5	Nuclear	3	1.99
Gene expression	Pp1s342_32V6.1	Histone h6	Nuclear	5	1.99
Gene expression	Pp1s165_12V6.2	Nucleoside diphosphate kinase	Cytoplasm	3	0.54
Gene expression	Pp1s77_158V6.2	GY-Box (GY)	Nuclear	4	0.27
Gluconeogenesis	Pp1s133_10V6.1	Phosphoenolpyruvate carboxykinase	Cytoplasm	2	1.67
Hydrolysis	Pp1s475_2V6.1	SucraseferRedoxin-like protein	Cytoplasm	2	0.41
Hydrolysis	Pp1s309_77V6.1	Glycoside hydrolase (chitinase)	Extracellular	3	0.40
Lipid metabolism	Pp1s434_27V6.1	Lipase/lipoxygenase, PLAT/LH2	Chloroplast	3	2.90
Lipid metabolism	Pp1s97_112V6.1	Cytochrome P450 (allene oxide synthase 2)	Chloroplast	4	2.22
Lipid metabolism	Pp1s8_168V6.1	Acetyl-biotin carboxylase	Chloroplast	14	1.96
Lipid metabolism	Pp1s18_23V6.1	Acetyl-biotin carboxylase	Chloroplast	12	1.90
Lipid metabolism	Pp1s419_7V6.1	Lipoxygenase	Chloroplast	26	1.53
Signaling	Pp1s348_15V6.1	14-3-3 Protein lil 1433-3	Nuclear	7	1.58
Signaling	Pp1s67_176V6.1	14-3-3 Protein lil 1433-3	Nuclear	7	1.58

Continued

Supplementary Table S1. Continued.

Function	Protein ID[1]	Description	Subcellular location	MP[2]	Ratio[3]
Signaling	Pp1s240_68V6.1	Signal-peptide (Unknown)	Extracellular	5	0.32
Stress	Pp1s91_109V6.1	Heat shock Protein, HSP70	Cytoplasm	3	1.76
Stress	Pp1s258_52V6.1	Heat shock Protein, HSP90	Cytoplasm	4	1.73
Stress	Pp1s291_62V6.1	Heat shock Protein, HSP90	Cytoplasm	11	1.61
Stress	Pp1s220_79V6.1	Heat shock Protein, HSP90	Cytoplasm	10	1.50
Stress	Pp1s220_83V6.1	Heat shock Protein, HSP90	Cytoplasm	10	1.50
Stress	Pp1s351_24V6.1	Heat shock Protein, HSP70	Cytoplasm	5	0.65
Stress	Pp1s61_17V6.4	S-formylglutathione hydrolase (esterase d)	Chloroplast	2	0.51
Stress	Pp1s153_153V6.2	Heat shock Protein, HSP70	Cytoplasm	2	0.39
Stress	Pp1s52_261V6.1	Late embryoGenesis abundant (plants) lea-related	Nuclear	2	0.25
Transport	Pp1s185_81V6.1	Non-green plastid inner envelope membrane Protein	Plasma Membrane	10	1.75
Transport	Pp1s85_94V6.1	Non-green plastid inner envelope membrane Protein	Plasma Membrane	9	1.68
Transport	Pp1s147_10V6.1	Clathrin heavy chain	Cytoplasm	6	1.57
Transport	Pp1s7_102V6.1	Clathrin heavy chain	Cytoplasm	6	1.57
Transport	Pp1s24_254V6.1	Unknown	Mitochondrial	4	0.59
Transport	Pp1s87_57V6.1	Clathrin light chain (expressed Protein)	Nuclear	2	0.37
Unknown	Pp1s125_81V6.5	Unknown	Cytoplasm	5	0.67
Unknown	Pp1s125_81V6.2	Unknown	Cytoplasm	6	0.54
Unknown	Pp1s200_89V6.1	Uncharacterized Protein family UPF0133	Chloroplast	2	0.44

[1]Protein IDs are from Phytozome ver. 11.0.9 (http://www.phytozome.net/).
[2]MP indicates the number of matched peptides.
[3]The ratio indicates the fold change between the control and wounding treatment group.

Supplementary Table S2. Proteins identified as responsive to wounding in the *aos* mutant of *P. patens*.

Function	Protein ID[1]	Description	Subcellular location	MP[2]	Ratio[3]
Protein synthesis	Pp1s172_22V6.1	Translation elongation factor EF1B	Extracellular	2	2.25
Protein synthesis	Pp1s311_58V6.1	Eukaryotic translation initiation factor	Cytoplasm	2	2.23
Protein synthesis	Pp1s215_81V6.1	Ribosomal Protein S13	Endoplasmic reticulum	3	2.22
Protein synthesis	Pp1s77_207V6.1	Ribosomal Protein L3	Nuclear	2	2.08
Protein synthesis	Pp1s215_71V6.1	Ribosomal Protein S13	Nuclear	2	2.08
Protein synthesis	Pp1s72_222V6.1	Ribosomal Protein S13	Nuclear	2	2.08
Protein synthesis	Pp1s39_223V6.2	Protein disulphide isomerase	Nuclear	2	2.08
Protein synthesis	Pp1s154_131V6.1	Ribosomal Protein L3	Nuclear	2	2.08
Protein synthesis	Pp1s127_74V6.1	Ribosomal Protein S3	Endoplasmic reticulum	3	2.05
Protein synthesis	Pp1s136_175V6.1	Ribosomal Protein S3	Nuclear	3	2.05
Protein synthesis	Pp1s315_40V6.1	Pre-pro-cysteine Proteinase	Chloroplast	2	1.88
Protein synthesis	Pp1s33_172V6.1	Leucyl Aminopeptidase-like Protein	Chloroplast	2	1.88
Protein synthesis	Pp1s215_81V6.1	Ribosomal Protein S13	Nuclear	3	1.85
Protein synthesis	Pp1s121_144V6.1	Ribosomal Protein L19/L19e	Cytoskeleton	2	1.84
Protein synthesis	Pp1s235_118V6.1	Ribosomal Protein L19/L19e	Cytoskeleton	2	1.84
Protein synthesis	Pp1s92_45V6.1	Ribosomal Protein S27e	Endoplasmic reticulum	3	1.64
Protein synthesis	Pp1s144_37V6.1	Ribosomal Protein L19/L19e	Endoplasmic reticulum	4	1.63
Protein synthesis	Pp1s218_59V6.1	Serine threonine-Protein kinase	Endoplasmic reticulum	4	1.63
Protein synthesis	Pp1s72_222V6.2	Ribosomal Protein S13	Chloroplast	3	1.59
Protein synthesis	Pp1s37_247V6.2	60s ribosomal Protein l2	Cytoplasm	4	1.50
Protein synthesis	Pp1s582_3V6.1	60s ribosomal Protein l2	Cytoplasm	4	1.50
Protein synthesis	Pp1s172_22V6.1	Translation elongation factor EF1B	Extracellular	2	2.25
Protein synthesis	Pp1s311_58V6.1	Eukaryotic translation initiation factor	Cytoplasm	2	2.23
Degradation	Pp1s109_234V6.1	26S proteasome subunit P45 (26s proteasome subunit 4)	Cytoplasm	2	2.59

Continued

Supplementary Table S2. Continued.

Function	Protein ID[1]	Description	Subcellular location	MP[2]	Ratio[3]
Degradation	Pp1s12_207V6.1	26S proteasome subunit P45 (26s proteasome subunit 4)	Cytoplasm	2	2.59
Degradation	Pp1s4_277V6.1	26S proteasome subunit P45 (26s proteasome subunit 4)	Cytoplasm	2	2.59
Degradation	Pp1s14_318V6.1	26S PROTEASOME NON-ATPASE REGULATORY S UBUNIT 4	Cytoplasm	2	2.07
Degradation	Pp1s285_10V6.1	Cysteine protease	Vacuolar	3	1.80
Degradation	Pp1s180_8V6.1	Neuromodulin (Growth-associated Protein 43)	Cytoplasm	3	1.75
Degradation	Pp1s152_13V6.1	26S protease regulatory subunit 7	Cytoplasm	4	1.62
Degradation	Pp1s49_256V6.1	26S protease regulatory subunit 7	Cytoplasm	4	1.62
Degradation	Pp1s72_282V6.1	Peptidase m16c associated domain Protein	Chloroplast	4	1.52
Degradation	Pp1s142_79V6.1	Pullulanase (a kind of glucanase)	Chloroplast	3	1.52
Amino acid metabolism	Pp1s350_23V6.2	Asparagine synthetase	Cytoplasm	2	2.05
Photosystem	Pp1s206_126V6.1	Germin-like Protein GLP2	Chloroplast	2	1.98
Photosystem II	Pp1s170_67V6.1	NADH:ubiquinone oxidoreductase, subunit G	Chloroplast	2	2.31
Photosystem II	Pp1s214_86V6.1	Type iii chlorophyll a b-binding Protein	Chloroplast	4	1.72
Photosystem II	Pp1s214_87V6.2	Type iii chlorophyll a b-binding Protein	Chloroplast	4	1.72
Photosystem II	Pp1s429_33V6.1	Type iii chlorophyll a b-binding Protein	Chloroplast	4	1.72
Glycolysis	Pp1s131_107V6.1	Sucrose-phosphate synthase	Cytoplasm	2	1.70
Glycolysis	Pp1s235_138V6.1	Lactate dehydrogenase (LDH)	Cytoplasm	2	1.62
Glycolysis	Pp1s333_15V6.1	Pyruvate kinase	Cytoplasm	2	1.51
Glycolysis	Pp1s70_15V6.1	Pyruvate kinase	Cytoplasm	2	1.51
Energy synthesis	NC_007945.1_cds id_YP_539003.1	ATPase subunit 8	Mitochondrial	2	1.82
TCA cycle	Pp1s26_26V6.1	Malate dehydrogenase	Mitochondrial	7	1.60
TCA cycle	Pp1s9_103V6.1	Succinate-CoA ligase	Mitochondrial	2	1.52
Redox	Pp1s106_16V6.1	NAD(P)-binding domain (formate dehydrogenase)	Cytoplasm	2	2.57

Continued

Supplementary Table S2. Continued.

Function	Protein ID[1]	Description	Subcellular location	MP[2]	Ratio[3]
Redox	Pp1s28_321V6.1	Formate dehydrogenase/DMSO reductase, domains 1-3	Cytoplasm	3	2.04
Redox	Pp1s95_65V6.1	Methionine sulfoxide reductase type	Chloroplast	2	1.89
Redox	Pp1s97_166V6.1	Methionine sulfoxide reductase type	Chloroplast	2	1.60
Redox	Pp1s40_48V6.3	Multicopper type 2(Multicopper oxidase, type 2&3)	Cytoplasm	4	1.60
Carbohydrate metabolism	Pp1s94_106V6.1	Pyrophosphate-fructose-6-phosphate 1-phosphotrans ferase (PFP)	Cytoplasm	3	1.52
Detoxification	Pp1s75_107V6.2	Glutathione S-transferase (glutathione s-transferase)	Chloroplast	2	3.06
Gene expression	Pp1s31_343V6.1	EUKARYOTIC TRANSLATION INITIATION FA CTOR 3F, EIF3F	Cytoplasm	2	2.54
Gene expression	Pp1s58_224V6.1	GY-Box (GY)	Nuclear	7	2.05
Gene expression	Pp1s140_60V6.1	Eukaryotic translation initiation factor 3	Cytoplasm	2	1.61
Hydrolysis	Pp1s6_50V6.1	Glycoside hydrolase, family 31	Vacuolar	2	2.18
Hydrolysis	Pp1s121_168V6.1	Glycoside hydrolase family	Chloroplast	4	1.67
Lipid metabolism	Pp1s181_57V6.4	Unknown	Vacuolar	4	1.70
Signaling	Pp1s159_85V6.2	SOUL haem-binding Protein (soul-like Protein)	Nuclear	2	1.81
Signaling	Pp1s44_58V6.1	GTP-binding Protein	Cytoplasm	3	1.73
Signaling	Pp1s84_187V6.1	Pirin-like Protein	Chloroplast	2	1.69
Signaling	Pp1s157_11V6.1	Glucan endo- -beta-glucosidase	Plasma Membrane	2	1.68
Signaling	Pp1s12_415V6.1	GRAM domain-containing Protein	Nuclear	7	1.51
Stress	Pp1s52_261V6.1	Late embryoGenesis abundant (plants) lea-related	Nuclear	2	4.10
Stress	Pp1s156_53V6.1	Germin-like Protein GLP4	Cytoplasm	4	2.15
Stress	Pp1s75_99V6.1	LATE EMBRYOGeneSIS ABUNDANT (LEA)	Nuclear	5	2.03
Stress	Pp1s9_107V6.2	12-OXOPHYTODIENOATE REDUCTASE OPR	Chloroplast	2	2.02
Stress	Pp1s86_31V6.1	Germin-like Protein (GLP4) (GLP5)	Cytoplasm	5	1.92
Stress	Pp1s326_44V6.1	12-oxophytodienoate reductase 2	Chloroplast	2	1.88

Continued

Supplementary Table S2. Continued.

Function	Protein ID[1]	Description	Subcellular location	MP[2]	Ratio[3]
Stress	Pp1s55_66V6.2	Late embryoGenesis abundant (plants) lea-related	Chloroplast	3	1.63
Stress	Pp1s82_6V6.1	Germin-like Protein (GLP4) (GLP5)	Extracellular	8	1.59
Stress	Pp1s55_65V6.1	Late embryoGenesis abundant (plants) lea-related	Chloroplast	2	1.57
Stress	Pp1s55_66V6.1	Late embryoGenesis abundant (plants) lea-related	Chloroplast	2	1.57
Stress	Pp1s59_239V6.1	Heat shock Protein, HSP70	Mitochondrial	9	1.54
Stress	Pp1s8_209V6.1	Heat shock Protein, HSP20	Chloroplast	2	1.53
Stress	Pp1s8_244V6.1	Heat shock Protein, HSP20	Chloroplast	2	1.53
Stress	Pp1s351_44V6.1	Seed maturation Protein	Plasma Membrane	2	1.53
Stress	Pp1s66_172V6.1	Glutathione s-transferase (GST)	Chloroplast	9	1.52
Transport	Pp1s252_67V6.2	Gene abcb16 multidrug resistance Protein	Plasma Membrane	7	1.95
Transport	Pp1s348_22V6.1	Plastid-Lipid-associated Protein	Chloroplast	4	1.77
Transport	Pp1s69_133V6.1	Putative Protein	Chloroplast	4	1.76
Transport	Pp1s2_600V6.1	Mitochondrial phosphate carrier Protein	Mitochondrial	3	1.58
Transport	Pp1s2_605V6.1	Mitochondrial phosphate carrier Protein	Mitochondrial	3	1.58
Transport	Pp1s86_72V6.2	Outer membrane lipoProtein blc	Chloroplast	4	1.58
Unknown	Pp1s306_59V6.1	Signal-peptide (Expressed Protein)	Vacuolar	2	4.68
Unknown	Pp1s335_17V6.1	MRO11.7; expressed Protein	Chloroplast	2	1.98
Unknown	Pp1s293_81V6.1	Unknown	Nuclear	9	1.97
Unknown	Pp1s13_231V6.1	Unknown	Nuclear	3	1.95
Unknown	Pp1s75_99V6.2	Unknown	Nuclear	4	1.82

[1]Protein IDs are from Phytozome ver. 11.0.9 (http://www.phytozome.net/).
[2]MP indicates the number of matched peptides.
[3]The ratio indicates the fold change between the control and wounding treatment group

Supplementary Table S3. List of primers used to construct the double-knockout mutant.

Primer name	Sequence
PpAOS1KO5’-F	5'-ATCTCGAGGGATCCCCATAGGAATAGG-3'
PpAOS1KO5’-R	5'-ATGAATTCGCTTGCCCAACACTACCA-3'
PpAOS1KO3’-F	5'-ATGCATGCGGAGTTCGTCTCCGAGAAC-3'
PpAOS1KO3’-R	5'-ATCATATGCACAACTTCACAGCCTCGTT-3'
PpAOS2KO5’-F	5'-ATAGGTACCAAGCCAGTAGATTGC-3'
PpAOS2KO5’-R.	5'-TATAAGCTTGCACAACACATTTGGC-3'
PpAOS2KO3’-F	5'-TAGCATGCAGGATTGGAGCAAGTG-3'
PpAOS2KO3’-R	5'-TGAGCTCGGTACCTCAAATCGAATCATG-3'
PpAOS1KO5’-F2	5'-TCAACGAATCCACAGAACGTGAAGTG-3'
PpAOS1KO3’-R2	5'-GCAACACCATATGCCATCACATC-3'
PpAOS2KO5’-F2	5'-AGAGCCAAGTTCGAAACAAGACTGCG-3'
PpAOS2KO3’-R2	5'-TGTTTGTAACACCATCCTTGCAGCG-3'
Pcmv-R	5'-GAGGAAGGGTCTTGCGAAGGATAGTG-3'
35SPA-F	5'-AGGAGGAAGACAAGGAAGGATAAGG-3'

Supplementary Table S4. List of primers used for qRT-PCR analyses.

Protein name	Protein ID	Primer name	Sequence
Chaperonin	Pp1s141_125V6.1	Pp_chap_1S	GCCGGAGTTTGAAGCCCTAT
		Pp_chap_1A	CATCTGCCACCGCCTTAGAG
Histone H4	Pp1s342_32V6.1	20190830-1-F	TATCACCAAGCCTGCCATCC
		20190830-1-R	TTAGCCTCCGAAACCATACAAAG
Ketol-acid reductoisomerase	Pp1s60_179V6.1	20190830-3-F	GAGGTGAGCACCCCAGGTAT
		20190830-3-R	CTTTTGAGCCGATTTTGAGC
PsbQ	Pp1s182_26V6.1	20190830-5-F	GAAGCTATTAAGGCTGTTAAGGATGT
		20190830-5-R	GCTGCGTAGTCCAAATTGTTCAG
PsbO	Pp1s306_84V6.1	20190830-6-F	AAGAGTTGGTGGCAGAAGGTAAA
		20190830-6-R	CTTGGCAATGCTGAAGGTGA
Pyruvate dehydrogenase e1 component subunit β	Pp1s156_57V6.1	20190830-7-F	ATGGTACGGCCAAGTTCCTG
		20190830-7-R	TCACTCCCTTCCCTCATTATCT
Malate dehydrogenase	Pp1s79_110V6.1	20190830-9-F	GGATGGCGACTCTGATGTTTAT
		20190830-9-R	AGACAGTGGCAGCTTGCTTG
ATP synthase β chain	Pp1s310_30V6.1	20190830-10-F	TTGAACACCGGGTCTCCTATC
		20190830-10-R	CGCCGAACAGTCCAATCTTAC
Actin	Ppactin-3U1*	Actin_F	CGGAGAGGAAGTACAGTGTGTGGA
		Actin_R	ACCAGCCGTTAGAATTGAGCCCAG

*The primers for actin are referenced in the following paper: Aoki S, Kato S, Ichikawa K, and Shimizu M. Circadian expression of the PpLhcb2 gene encoding a major light-harvesting chlorophyll a/b-binding protein in the moss *Physcomitrella patens*. *Plant Cell Physiol.*, 45, 68–76 (2004).
